# Dynamic Atlas and Energy Metabolism Adaptation of the Liver Proteome During a 24 h Cycle in *Vespertilio sinensis*

**DOI:** 10.3390/biom16091289

**Published:** 2026-09-06

**Authors:** Tianhui Wang, Yujia Chu, Xin Li, Haokun Zheng, Jiang Feng, Hui Wang

**Affiliations:** 1 College of Life Science, Jilin Agricultural University, Changchun 130118, China; wangtianhui@mails.jlau.edu.cn (T.W.); chuyujia@mails.jlau.edu.cn (Y.C.); lixin@mails.jlau.edu.cn (X.L.); 20240902@mails.jlau.edu.cn (H.Z.); 2 Jilin Provincial International Cooperation Key Laboratory for Biological Control of Agricultural Pests, Jilin Agricultural University, Changchun 130118, China; 3 Jilin Provincial Key Laboratory of Animal Resource and Ecological Security, Northeast Normal University, Changchun 130117, China

**Keywords:** bats, liver, circadian rhythm, proteomic, multi-omics, metabolic adaptation

## Abstract

Circadian rhythms regulate hepatic metabolism and physiological homeostasis, which are essential for organisms to adapt to environmental cycles. Bats are the only mammals capable of sustained powered flight, accompanied by extreme metabolic demands and a nocturnal lifestyle. However, the regulatory mechanisms underlying hepatic circadian rhythms in bats remain poorly understood. Herein, we employed data-independent acquisition (DIA)-based quantitative proteomics to characterize the diurnal dynamics of hepatic proteins in *Vespertilio sinensis* across four representative physiological states, and further conducted integrative multi-omics analysis combined with transcriptomic data. The results revealed that approximately 5% of hepatic proteins exhibited circadian rhythmicity in *V. sinensis*, a proportion markedly higher than that of rhythmic transcripts and metabolites. Prominent transcription-protein rhythm decoupling was observed, implying that post-transcriptional regulatory processes may play critical roles in shaping circadian rhythms. Core metabolic pathways displayed temporally dynamic enrichment. The diurnal functional switching of the liver was achieved via stable expression of core proteins and temporal turnover of specific proteins, which efficiently coordinated energy supply, oxidative defense and detoxification metabolism. Trend clustering and functional enrichment analyses suggested that the liver may undergo lysosome-mediated immune repair during the daytime, while exhibiting enriched energy metabolic profiles that potentially support the elevated energy expenditure required for flight at night. This study systematically elucidates the protein regulatory patterns underlying hepatic diurnal rhythms in *V. sinensis*, and provides molecular clues for exploring extreme energy adaptation and circadian evolutionary scenarios in nocturnal flying mammals.

## 1. Introduction

Circadian rhythm is a fundamental biological trait evolved by organisms to adapt to the day–night alternation caused by Earth’s rotation. It precisely modulates diverse physiological processes and behavioral patterns at both systemic and cellular levels [1,2]. The core circadian clock is driven by transcription–translation negative feedback loops composed of key clock genes such as BMAL1/CLOCK and PER/CRY [3]. By regulating the periodic expression of thousands of downstream rhythmic genes, it temporally orchestrates vital activities including metabolism, immunity and cell proliferation in a precise manner [4]. The liver is a vital metabolic organ in mammals and indispensable for maintaining systemic energy homeostasis. Hepatic metabolic processes dominated by gluconeogenesis, glycogen metabolism, lipid synthesis and fatty acid oxidation are all tightly controlled by the circadian clock. Such regulation enables the liver to adapt to diurnal cycles of feeding and fasting, activity and sleep, thereby sustaining whole-body metabolic homeostasis [5,6]. Accumulating evidence has demonstrated that disruption of hepatic metabolic rhythms directly leads to a variety of metabolic disorders, such as insulin resistance, fatty liver disease and metabolic syndrome. This highlights the central role of rhythmic metabolic regulation in the maintenance of organismal homeostasis [7,8].

Previous studies on hepatic metabolic rhythms have relied on gene expression microarrays or RNA sequencing (RNA-seq), which have successfully identified a large number of transcripts with circadian oscillatory patterns [9]. Nevertheless, recent multi-omics studies using model organisms such as mice have revealed prominent phase separation or temporal lag between mRNA abundance and protein expression levels [10,11]. As proteins serve as the ultimate executors of physiological functions and metabolic reactions, post-transcriptional modification, translation efficiency and the protein degradation rate collectively determine the activity of metabolic enzymes and the implementation of physiological rhythms [12]. Accordingly, exploring hepatic circadian rhythms at the proteomic level is critical to fully uncover the execution mechanisms of hepatic metabolic rhythms and post-transcriptional buffering. This research strategy adopts quantitative proteomics based on data-independent acquisition (DIA), a technique characterized by high throughput and high sensitivity [13].

Bats are the only group of mammals that have evolved the capacity for sustained powered flight. This unique locomotive ability confers strong ecological adaptability yet also imposes extreme challenges on energy metabolism [14,15]. Their metabolic rate during flight can reach 10–20 times the resting level, far exceeding that of terrestrial mammals during locomotion. This places stringent demands on energy supply and the balance of oxidative stress [16]. To sustain long-distance flight and foraging at night, bats experience a sharp rise in their metabolic rate during active phases. In contrast, they undergo prolonged sleep and fasting throughout the daytime. Such an extreme cycle of feeding–fasting and activity–sleep necessitates an extremely efficient and flexible mechanism for switching metabolic rhythms in the bat liver, enabling rapid mobilization of energy reserves and prompt restoration of physiological homeostasis.

Despite the great research value of bats in metabolic adaptation, antiviral innate immunity and anti-aging longevity, the dynamic patterns of rhythmic proteins in bat liver and their adaptive mechanisms underlying extreme ecological traits remain largely unclear. In the present study, wild *Vespertilio sinensis* was selected as the research subject. We adopted high-sensitivity DIA-based quantitative proteomics to systematically characterize hepatic proteome dynamics across four typical physiological states within a diurnal cycle: satiation, sleep, fasting and activity. This study aims to clarify the metabolic responses during the sequential transition among the four states. Combined with previously acquired hepatic transcriptomic data [17], we further explored the diurnal patterns of key rhythmic proteins and related physiological processes, and revealed the coordination and divergence between gene and protein rhythms in bat liver. This work not only systematically elucidates the diurnal dynamics of the hepatic proteome in bats, but also provides valuable resources for further dissection of molecular mechanisms potentially linked to extreme energy metabolism adaptation.

## 2. Materials and Methods

### 2.1. Sample Collection

Sample collection was conducted at the natural roosts of *V. sinensis* in Acheng District, Harbin, Heilongjiang Province, from July to August 2022. Prior to formal sample collection, seven consecutive days of continuous field behavioral observation were performed on wild *V. sinensis* population to record their diurnal feeding, resting and flight rhythms. Four representative time points corresponding to the peak stable plateau of distinct physiological cycles were selected: 04:00 (satiation), 10:00 (sleep), 16:00 (fasting), and 22:00 (activity). Each physiological behavioral state persists for several hours with stable phenotypic characteristics, and all sampling time points fall within these steady windows. Restricted by wildlife protection requirements and the risk of stress-induced physiological damage to bats from repeated collection, samples were only collected at one single characteristic time point within each physiological plateau. This sampling strategy enables us to capture the typical hepatic proteomic signatures of each physiological state.

All sampling work was completed within a single 24 h period to preserve the bats’ native wild physiological state. Bats were collected sequentially according to predefined sampling time points. On the sampling day, the local sunrise was at 04:07 and sunset at 19:09, with a natural daylight duration of 15 h and 2 min. The ambient temperature ranged from 20 °C to 27 °C during the day. No laboratory acclimation or artificial light–dark cycle manipulation was conducted. All sampled bats were non-lactating adult females, which minimizes individual variation arising from gender and developmental stage. For the states of satiation, sleep, and fasting, bats were captured by hand-draft nets, and for the active state, bats were captured by mist nets. Immediately after capture, individuals were humanely euthanized by cervical dislocation to minimize animal distress. At each time point, liver tissues were randomly collected from three non-lactating adult females under similar environmental conditions to serve as biological replicates. A total of 12 individuals were included for subsequent sequencing analysis. After capture, body temperature, body weight and forearm length of each bat were measured immediately. All tissue dissections were carried out by one single researcher in strict accordance with a unified standardized protocol to reduce variability in post-mortem intervals and ensure consistent protein quantification across all samples. The liver tissues were then dissected and placed into sterile cryogenic tubes, followed by flash freezing in liquid nitrogen overnight. Once all samples were collected, they were transported to the laboratory on dry ice and stored in an ultra-low-temperature freezer at −80 °C.

### 2.2. Protein Extraction and Quantification

Each bat liver tissue sample was ground in liquid nitrogen, and approximately 0.2 g of the ground tissue was used for protein extraction. Then, 600 µL of lysis buffer containing 4% SDS and phenylmethylsulfonyl fluoride (PMSF, a protease inhibitor) was added to each sample, followed by thorough grinding for 10 min. The samples were centrifuged at 13,000× *g* and 4 °C for 10 min, and incubated in a dry bath at 90 °C for 10 min. The centrifugation and heating steps were repeated once. After another round of centrifugation, the clear supernatant containing proteins was collected.

Protein quantification was performed using the BCA assay. A standard curve was generated with BSA standards ranging from 0 to 50 µg. Samples were incubated with BCA working solution at 37 °C for 30 min in the dark, and absorbance was measured at 562 nm to calculate protein concentrations. For quality assessment, 30 µg of protein from each sample was subjected to SDS-PAGE at 200 V for 30 min, followed by Coomassie brilliant blue staining.

### 2.3. Protein Digestion (FASP) and High-pH Reverse-Phase Fractionation (HPLC)

A total of 100 µg protein was taken from each sample and digested using the FASP method [18]. The volume was adjusted to 200 µL with 8 M urea. DTT was added to a final concentration of 10 mM, and the mixture was incubated at 56 °C for 30 min. IAA was then supplemented to a final concentration of 50 mM, followed by incubation at room temperature for 40 min in the dark. The mixture was transferred into a 10 kDa ultrafiltration tube and centrifuged at 12,000× *g* at room temperature, after which the filtrate was discarded. The retentate was washed three times with 8 M urea and then three times with 50 mM ammonium bicarbonate solution. Trypsin was added at a mass ratio of 1:50 (enzyme to protein, m/m). The sample was incubated at 37 °C for 16 h for enzymatic digestion. The filtrate containing peptides was collected by centrifugation at 12,000× *g* and 4 °C, and lyophilized for subsequent analysis.

A portion of the digested peptides was fractionated via high-pH reversed-phase chromatography using an Agilent 1100 HPLC system equipped with a Waters XBridge C18 column (5 µm, 4.6 mm × 250 mm; Waters Corporation, Milford, MA, USA). Mobile phase A consisted of 98% water and 2% acetonitrile (ACN, pH 10), and mobile phase B consisted of 98% ACN and 2% water (pH 10). A 60 min linear gradient elution was performed as follows: 0–5 min, 3% B; 5–10 min, 5% B; 10–35 min, 18% B; 35–45 min, 34% B; 45–58 min, 95% B; 58–60 min, 3% B. The flow rate was set at 0.4–0.7 mL/min. Fractions were collected at one fraction per minute. After removing the flow-through peak, the collected fractions were combined in a concatenation pattern for subsequent DDA library construction.

### 2.4. LC-MS/MS Analysis

The three pooled fractions obtained from fractionation were subjected to data-dependent acquisition (DDA) for database construction. All formal samples were analyzed in DIA mode, with one injection per sample. Protein quantification was subsequently performed by searching DIA datasets against the DDA-derived database. For liquid chromatography (LC), the flow rate was set to 600 nl/min and the injection volume was 6 µL. For mass spectrometry (MS), analyses were operated in positive ion mode with a spray voltage of 2.2 kV and probe temperature of 320 °C. The full scan mass range was set from 350 to 1500 m/z.

### 2.5. Database Construction and Quality Control

Proteome Discoverer 2.1 (Thermo Fisher Scientific, Rockford, IL, USA) was used to establish the DDA database for subsequent DIA quantification. Database searching was performed with the Sequest HT engine [19] against the UniProt database (https://www.uniprot.org/ (accessed on 15 August 2022)). A target-decoy search strategy was applied to control false positives, with the false discovery rate (FDR) ≤ 0.01 at the peptide level and *q* value ≤ 0.01 at the protein level as the cut-off criteria. Key parameters for DDA database construction were set as follows. Trypsin was specified as the digestion enzyme, and up to two missed cleavage sites were allowed. Variable modifications included protein N-terminal acetylation and deamidation of asparagine and glutamine (N, Q). Carbamidomethylation of cysteine (C) was defined as a fixed modification. The mass tolerance was set to ±10 ppm for precursor ions and ± 0.02 Da for fragment ions. Both peptide FDR and protein *q* value were strictly restricted below 1%.

### 2.6. DIA Quantification and Protein Functional Annotation

Spectronaut software [20] was used for protein identification and quantification of raw DIA data. The reference database was generated from DDA data using Proteome Discoverer 2.1 (Thermo Fisher Scientific, Waltham, MA, USA). By matching raw DIA data against the reference database, the chromatographic peak areas of unique peptides were extracted to represent relative protein abundance for expression comparison across samples. To ensure quantification accuracy, strict filtering criteria were applied to quantified peptides and fragment ions. Peptide length was restricted to 6–25 amino acids. Only b- and y-type fragment ions with m/z values higher than precursor ions were retained, while the last three fragment ions were excluded. Each peptide was assigned 2 to 5 fragment ions. The ion extraction window was set to 5 min, and the Dotp threshold was no less than 0.6. To resolve peptide-to-protein inference ambiguity during spectral library construction, shared peptides were assigned to protein groups instead of individual protein entries. Only unique peptides were used to calculate relative abundance at the protein-group level, whereas shared peptides were retained for peptide-spectrum-match-based identification but not incorporated into protein-group intensity calculations.

Proteins identified by mass spectrometry were annotated by searching against multiple public databases, including CAZy (https://www.cazy.org/ (accessed on 15 August 2022)), GO (http://www.geneontology.org/ (accessed on 15 August 2022)), KOG (https://ftp.ncbi.nlm.nih.gov/pub/COG/KOG/ (accessed on 15 August 2022)) and KEGG (http://www.kegg.jp/ (accessed on 15 August 2022)). For further in-depth functional analysis, protein domain prediction was performed using the Pfam database. In addition, subcellular localization of proteins was predicted using WoLF PSORT software [21] to infer their potential biological functional sites.

### 2.7. Identification of Differentially Expressed Proteins (DEPs) and Functional Enrichment Analysis

Raw expression values were normalized to eliminate deviations caused by sequencing depth. The *p* values were calculated using a statistical model, and the Benjamini–Hochberg (BH) method was applied for multiple-testing correction to obtain the FDR. Differential expression analysis of proteins was performed using mapDIA software [22]. To comprehensively capture proteomic shifts occurring during every physiological transition throughout the diurnal cycle, we performed all possible pairwise comparisons between the four states, generating six independent comparison groups: satiation vs. sleep, sleep vs. fasting, fasting vs. activity, activity vs. satiation, satiation vs. fasting, and sleep vs. activity. This full pairwise comparison strategy avoids missing expression changes that may only emerge during specific state transitions, rather than limiting analysis to adjacent sequential time points only. The screening criteria for DEPs were set as |log_2_ fold change| ≥ 1 and *p* < 0.05; BH-corrected *q* values were also provided for reference. Subsequently, GO functional enrichment and KEGG pathway enrichment analyses were separately carried out for DEPs from the six comparison groups. These two enrichment analyses were performed based on the custom background set consisting of all proteins quantitatively detected in this study. Enrichment significance was determined using Fisher’s exact test, with statistical thresholds applied to identify significantly enriched functional terms. GO terms with *p* < 0.05 and KEGG pathways with adjusted *p* < 0.05 were defined as significantly enriched items.

### 2.8. Trend Analysis and Rhythmicity Analysis

To clarify the diurnal temporal patterns of protein expression in the liver of *V. sinensis*, we compiled the union of all DEPs identified via pairwise comparisons across the four physiological time points. Based on the corresponding quantitative expression matrix, short time-series expression clustering was performed using the STEM (Short Time-series Expression Miner) program. Modules with significant expression trends were screened at *p* < 0.05. Proteins clustered into these significant modules were considered to share similar temporal expression patterns. GO functional annotation and KEGG pathway enrichment analyses were further conducted for proteins within the significant trend modules, with *p* < 0.05 set as the threshold to identify significantly enriched terms and pathways, so as to explore their associated biological functions and metabolic processes.

To systematically identify rhythmically expressed proteins and characterize their functional patterns in bat liver, rhythmicity analysis was performed on all quantifiable proteins using the Cosinor algorithm implemented in the DiscoRhythm package. The oscillation period was fixed at 24 h. Raw *p* values were subjected to BH correction to obtain *q* values. Considering the limited temporal sampling, raw *p* < 0.05 was used as the threshold for identifying rhythmic proteins, while *q* values were reported for reference. Amplitude and acrophase were derived as fitted descriptive parameters and were not used for filtering. For each time point, protein abundances were averaged across biological replicates prior to Cosinor fitting.

### 2.9. Integrative Analysis of Transcriptome and Proteome Data

To explore the correlation between gene transcription and protein translation in the liver of *V. sinensis*, we performed integrative comparison of transcriptomic and proteomic datasets. Given the absence of a reference genome for *V. sinensis*, *de novo* assembled transcripts were annotated via BLAST (version 2.13.0) against the reference proteome of closely related bat species (*Pipistrellus kuhlii*, GCF_014108245.1) to obtain gene name and UniProt-ID annotations. Identified proteins from proteomic data were also annotated with gene names and UniProt IDs. Gene–protein relationships were established by matching these annotated gene names and UniProt IDs. For genes with multiple aliases, identifiers were standardized to gene names from the reference bat species. The quantities of genes corresponding to total proteins, rhythmic proteins and DEPs were statistically analyzed. Gene–protein pairs with consistent rhythmic expression patterns were further screened. A correlation network of differentially expressed genes (DEGs) and DEPs was constructed based on their expression matrices. Expression correlations between genes and proteins were calculated to identify hub proteins serving as key nodes within the network. Network visualization was conducted via Cytoscape software (version 3.9.1).

## 3. Results

### 3.1. Proteome Identification, Domain Composition and Subcellular Localization Characteristics

**Figure 1:**
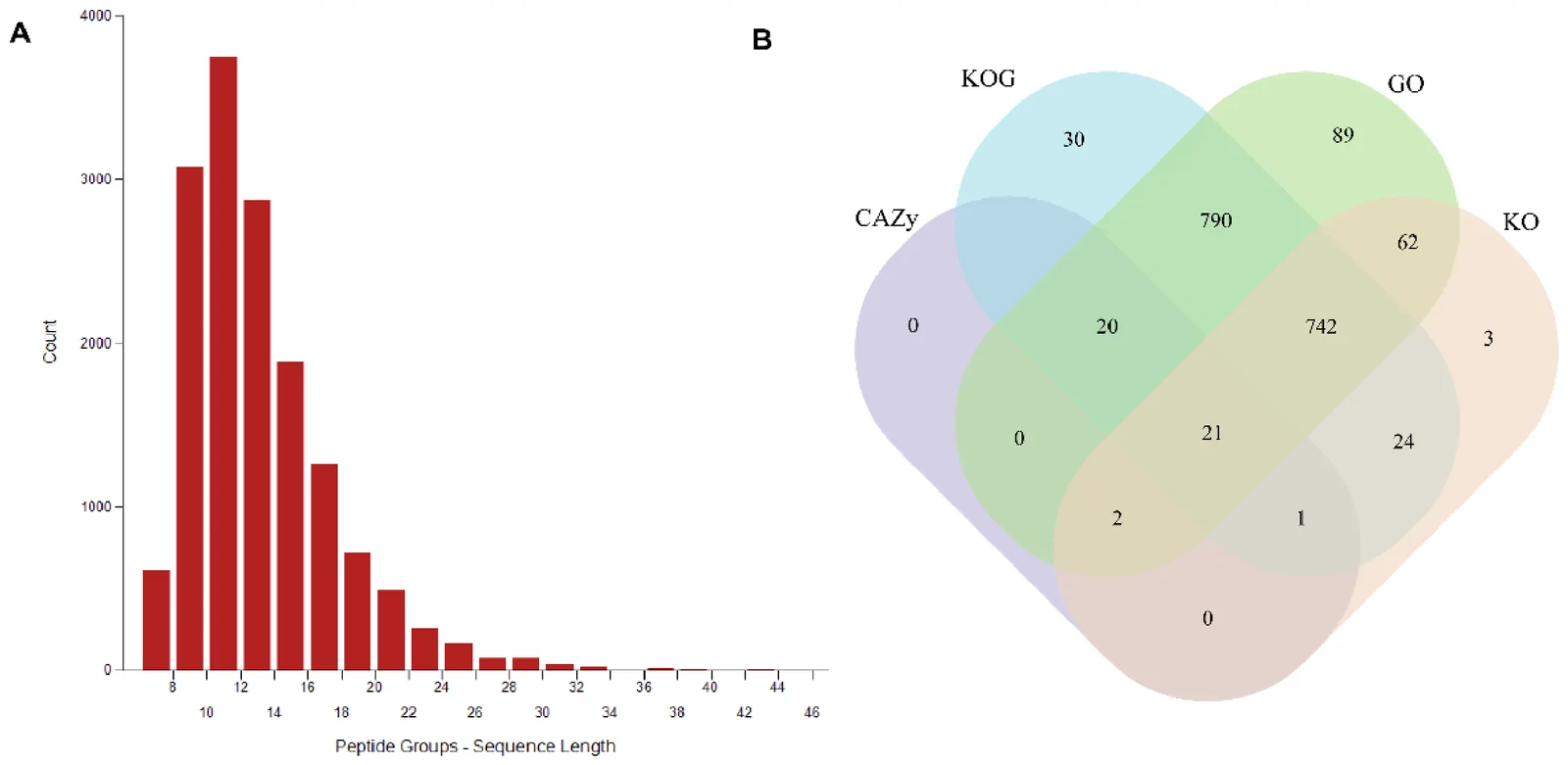
Distribution of peptide amino acid lengths (**A**) and Venn diagram of proteome annotation results across different databases (**B**). The peptide-length distribution in (**A**) was generated based on unique peptides.

In this wild-bat sampling design, circadian time, feeding status and behavioral activity are inherently confounded and their independent effects cannot be disentangled in the present study. The four sampling time points correspond to distinct physiological phases (satiation, sleep, fasting, and activity). Accordingly, we only describe molecular profiles observed within each sampling time-window and refrain from assigning causal effects to any single physiological variable. Herein, quantitative proteomic analysis was performed on liver tissues from 12 *V. sinensis* individuals across four consecutive physiological states, including satiation, sleep, fasting and activity, using a mass spectrometry-based DIA approach. Quality control results revealed that most peptides ranged from 6 to 25 amino acids in length, with the longest peptide containing 44 amino acids. The majority of peptides had no missed cleavage sites (Figure 1A), indicating satisfactory protein digestion efficiency and reliable data quality. Principal component analysis (PCA) showed that biological replicates from the same physiological state tended to group together. PC1 and PC2 explained 30.29% and 17.04% of the total variance, respectively. A total of 1803 proteins were identified in the present work. Among them, 1726, 1628, 855 and 44 proteins were annotated against the GO, KOG, KEGG and CAZy databases, respectively (Figure 1B).

**Figure 2:**
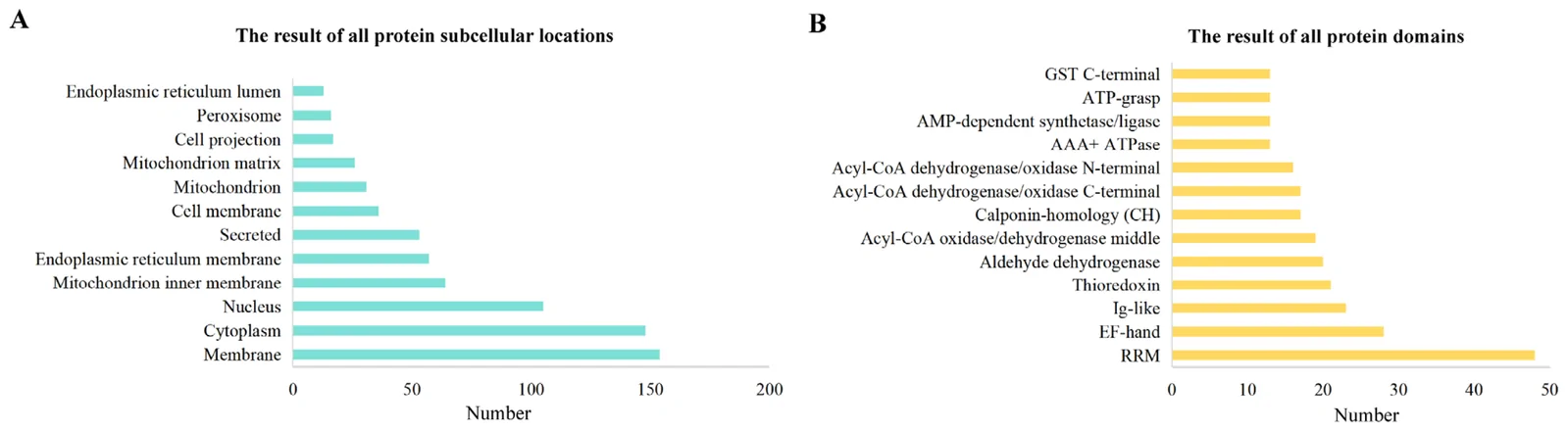
Protein subcellular localization (**A**) and protein domain (**B**).

Subcellular localization analysis of the identified liver proteins showed that most proteins were distributed in the membrane, cytoplasm and nucleus, followed by the mitochondrion inner membrane, endoplasmic reticulum membrane, and secreted. Proteins were also detected in subcellular structures including the mitochondrial matrix and peroxisomes. These subcellular assignments stem from computational predictions and lack experimental verification. Protein spatial distribution could be dynamically reshaped by protein–protein interactions and shifting physiological states, so these distribution trends should be considered with appropriate caution. Conserved domain analysis indicated that RRM, EF-hand, Ig-like and thioredoxin domains had the highest abundance. Domains associated with aldehyde dehydrogenase and Acyl-CoA oxidase/dehydrogenase middle were also widely distributed (Figure 2).

### 3.2. Analysis and Enrichment Characteristics of DEPs in Bat Liver

**Figure 3:**
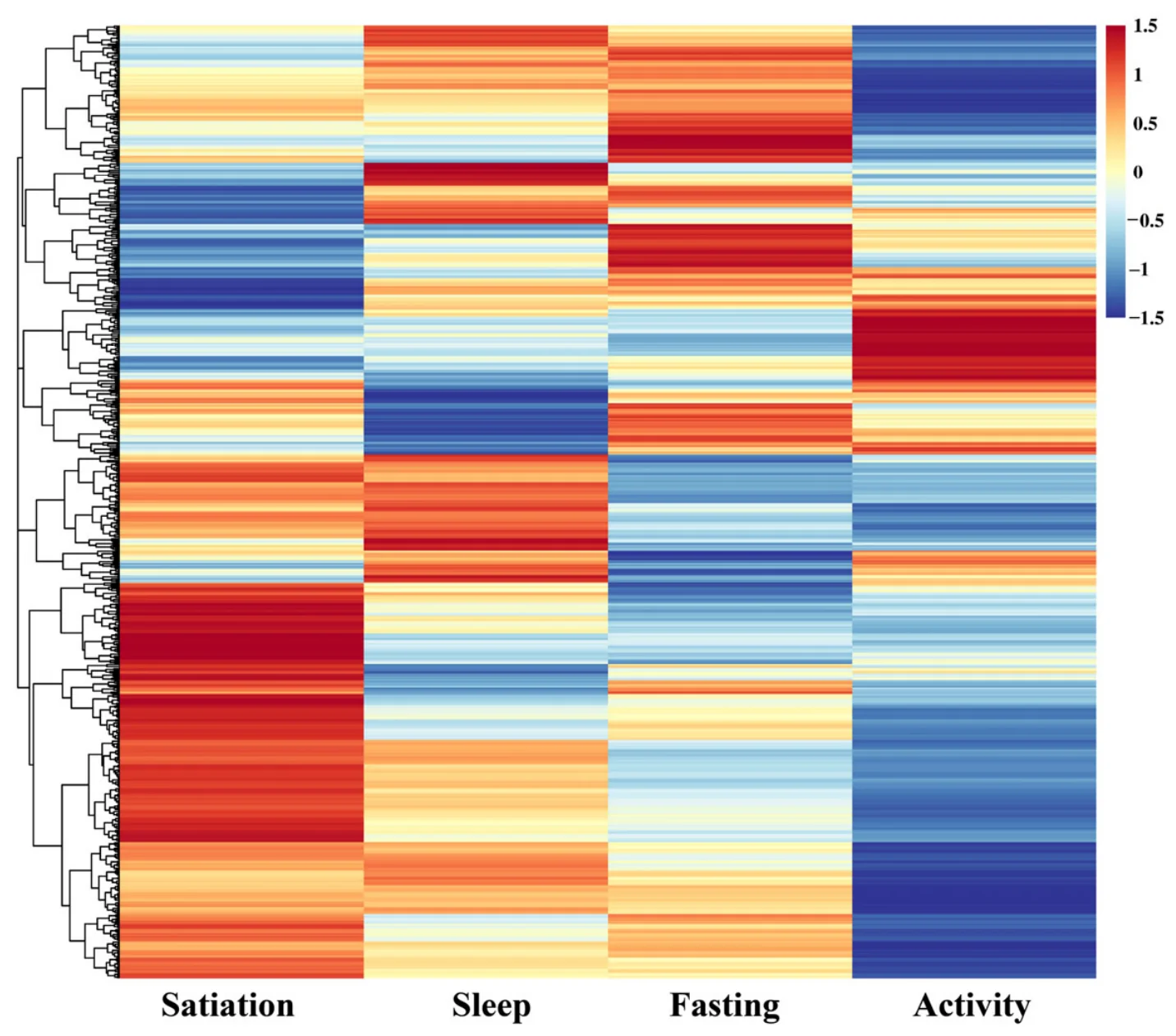
Heatmap showing the abundance of liver DEPs across four physiological states. Each physiological condition is represented by samples collected at a single discrete time point. The rationale for state definition and sampling design are detailed in Materials and Methods (Section 2.1).

To explore the overall expression differences in liver proteins across the four physiological states, DEPs were screened from six pairwise comparison groups: satiation vs. sleep, sleep vs. fasting, fasting vs. activity, activity vs. satiation, satiation vs. fasting, and sleep vs. activity. There were 163, 172, 241, 402, 244 and 296 DEPs identified for each group, respectively (Table 1 and Table S1). An up-regulated protein was defined as one that had a higher expression level in the former state than in the latter and vice versa for a down-regulated protein. The union of DEPs from all six groups yielded 692 unique DEPs. The expression variations of these proteins among different physiological states are presented in Figure 3.

**Figure 4:**
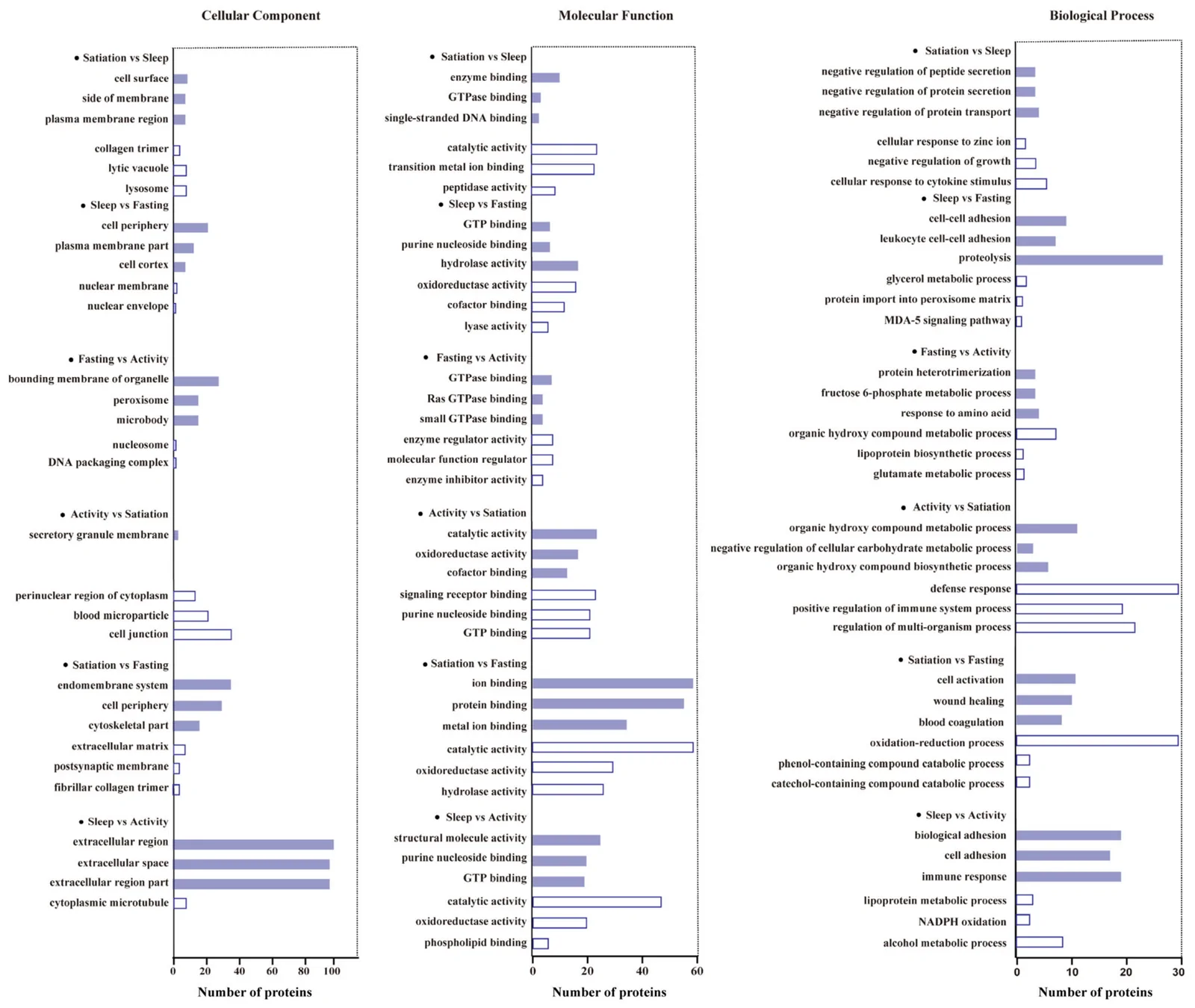
GO terms significantly enriched by DEPs from six comparison groups (full results in Table S2). Up to three significantly enriched terms are displayed. Solid and hollow bars represent terms enriched by up-regulated and down-regulated proteins, respectively. Note that enriched terms include both group-specific signatures and shared terms detected across multiple comparisons. DEPs within each comparison group do not require synchronized circadian rhythmicity and may display divergent rhythmic phases.

GO functional enrichment analysis was performed on DEPs from the six comparison groups, and the results are shown in Table S2 and Figure 4. Taking the satiation vs. sleep group as an example, at the cellular component level, proteins with higher abundance in the satiation state were significantly enriched in terms such as cell surface (GO:0009986) and side of membrane (GO:0098552), while highly abundant proteins in the sleep state were enriched in collagen trimer (GO:0005581) and lytic vacuole (GO:0000323). At the molecular functional level, highly abundant proteins in the satiation state were significantly enriched in terms including enzyme binding (GO:0019899) and GTPase binding (GO:0051020). Those highly expressed during sleep were significantly enriched in catalytic activity (GO:0003824) and transition metal ion binding (GO:0046914). For biological processes, abundant proteins in the satiation state were enriched in negative regulation of peptide secretion (GO:0002792) and negative regulation of protein secretion (GO:0050709). By contrast, highly expressed proteins in the sleep state were enriched in cellular response to zinc ion (GO:0071294) and negative regulation of growth (GO:0045926).

**Figure 5:**
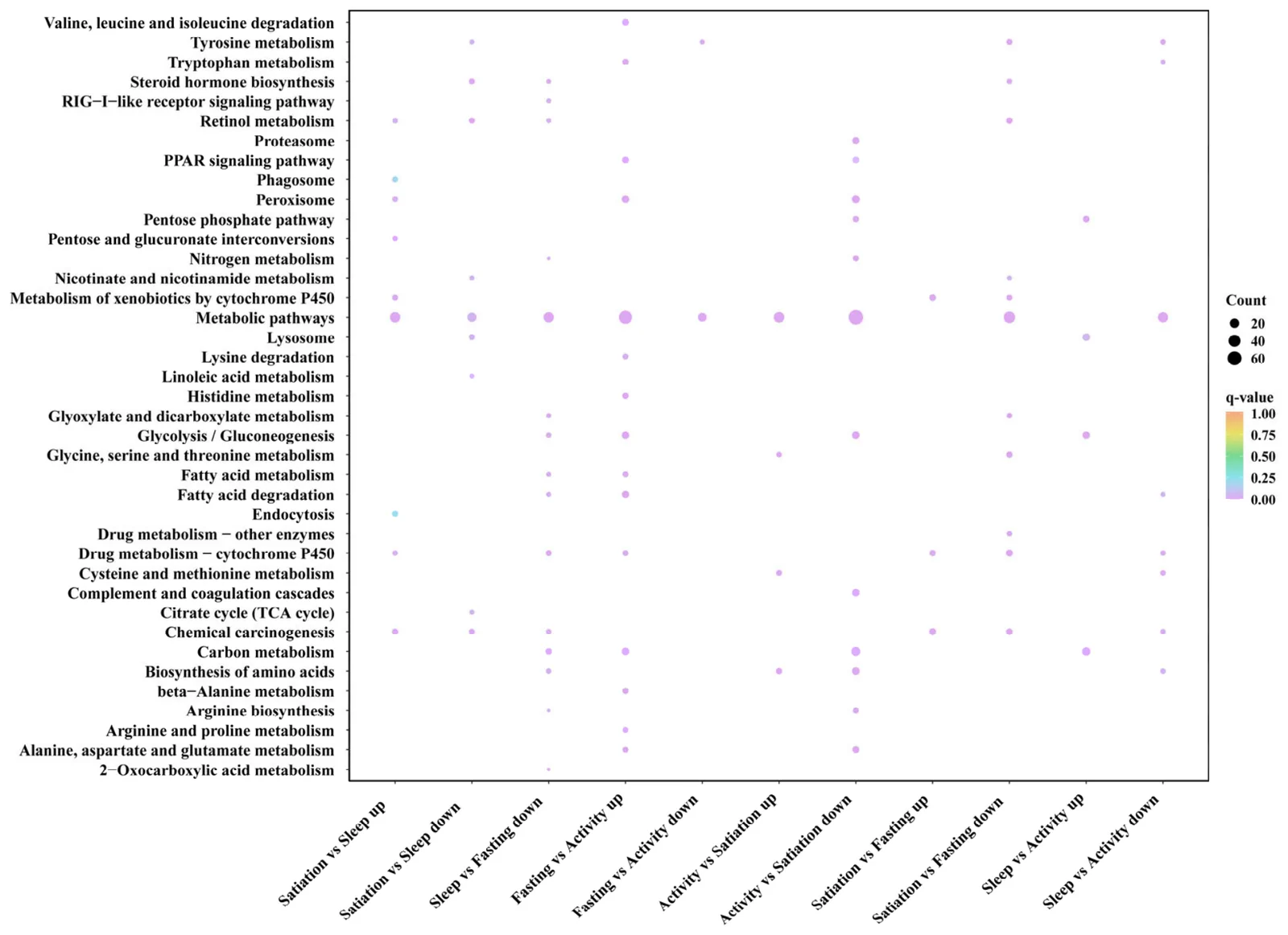
KEGG pathways significantly enriched by DEPs from six comparison groups (full results in Table S3). Notes as for Figure 4.

KEGG pathway enrichment analysis was conducted for up-regulated and down-regulated proteins from the six pairwise comparison groups. The results (Table S3, Figure 5) revealed that multiple pathways were consistently co-enriched across different physiological states.

**Figure 6:**
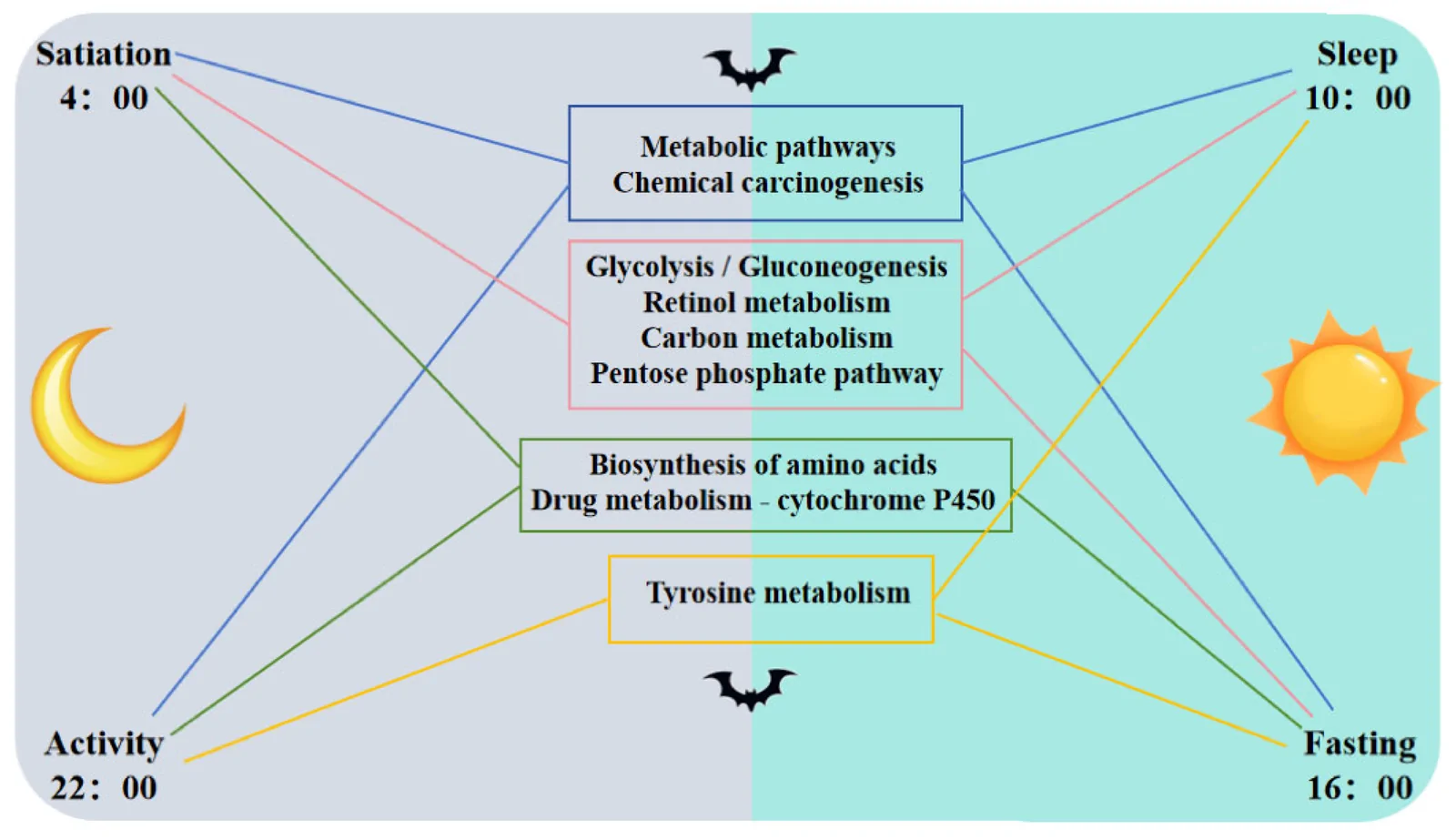
Temporal characteristics of core pathways across different physiological stages (summarized from Table S3).

Metabolic pathways (ko01100) and Chemical carcinogenesis (ko05204) were significantly enriched across all four physiological states, serving as the core pathways supporting diurnal physiological activities in bat liver. Glycolysis/gluconeogenesis (ko00010), Retinol metabolism (ko00830), Carbon metabolism (ko01200) and the pentose phosphate pathway (ko00030) were co-enriched in the satiation, sleep and fasting states. Biosynthesis of amino acids (ko01230) and Drug metabolism-cytochrome P450 (ko00982) were synchronously enriched in satiation, fasting and activity states. Tyrosine metabolism (ko00350) showed consistent enrichment during sleep, fasting and activity (Figure 6). These commonly enriched pathways across multiple physiological states persist throughout the diurnal cycle of bats. They are suggested to participate in the rhythmic regulation of hepatic energy metabolism, substance synthesis and detoxification homeostasis, and act as key pathways associated with diurnal metabolic regulation in the liver.

Further analysis indicated that although several KEGG pathways were significantly enriched across multiple physiological states, they contained both highly abundant proteins shared by all states and state-specific DEPs. Taking Glycolysis/gluconeogenesis as an example, this pathway was significantly enriched in satiation, sleep and fasting states. ACSS1, FBP1, GPI and PFKL were highly abundant in all three states and maintained the basic functions of the pathway. Meanwhile, distinct proteins were specifically enriched in each state: PDHB and FBP2 were predominant in the satiation state; PCK2 was specifically expressed during sleep; and ADH7, ALDH3A1, DLAT, GAPDH and ALDH2 showed high abundance exclusively in the fasting state. Collectively, the conserved core proteins sustain the fundamental metabolic functions of the pathway, while the temporal alternation of state-specific enzymes enables fine-tuned rhythmic regulation of pathway activity.

### 3.3. Protein Expression Trend Clustering Results

**Figure 7:**
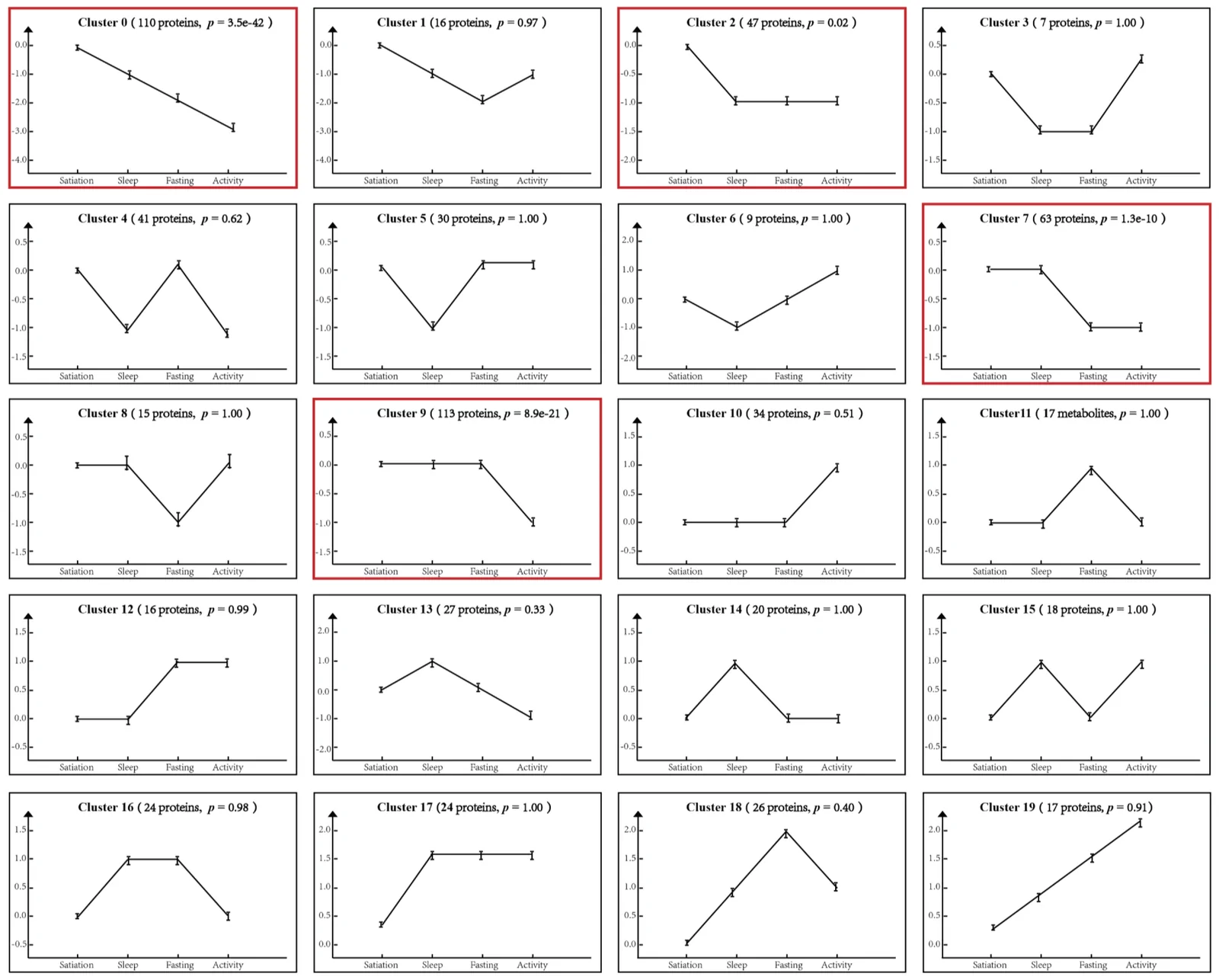
Trend clusters of all DEPs; the four clusters with significant protein clustering are outlined in red.

Trend analysis revealed that the 692 DEPs were grouped into 20 distinct temporal expression patterns (Figure 7). Four clusters (Cluster 0, Cluster 2, Cluster 7 And Cluster 9) were identified as statistically significant, containing 110, 47, 63 and 113 proteins respectively.

**Figure 8:**
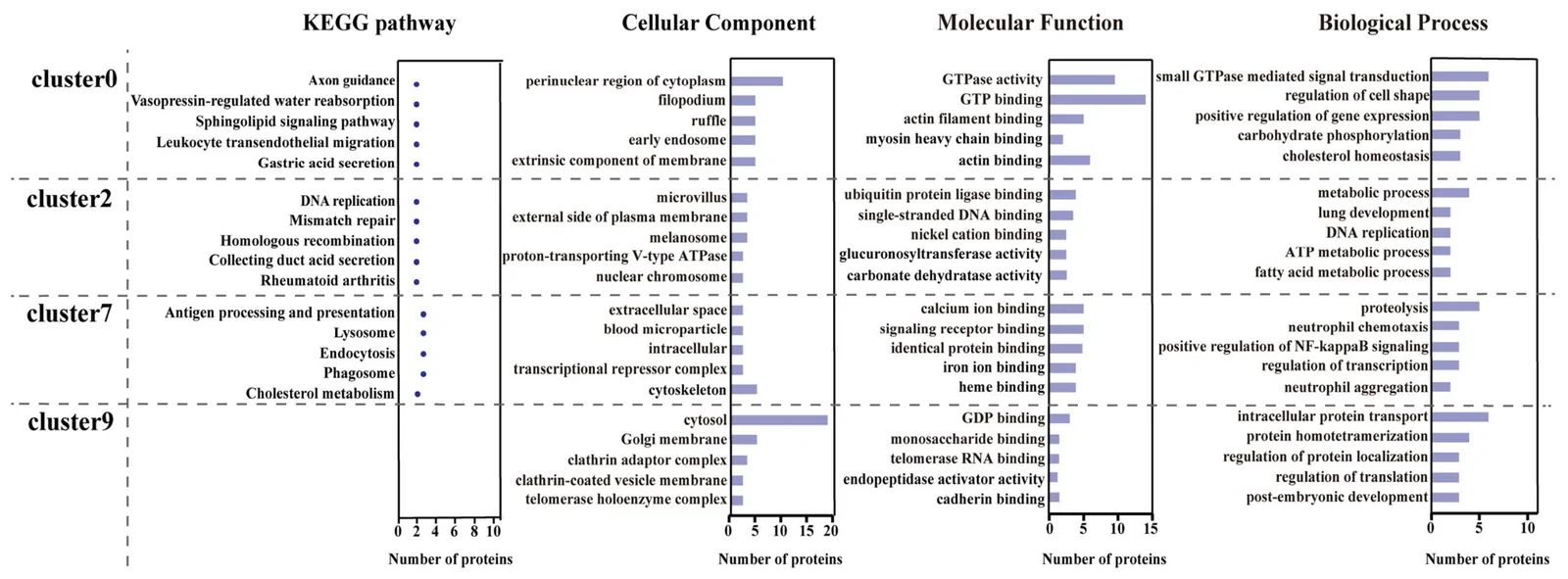
Functional enrichment results of proteins from the four significant trend clusters (full results in Table S4). Only the top five significant GO terms or KEGG pathways are presented.

Functional enrichment analysis was performed on proteins from the four significant trend clusters, with results presented in Table S4 and Figure 8. The protein abundance in Cluster 0 decreased continuously from satiation to activity. These proteins were mainly enriched in terms such as GTPase activity (GO:0003924) and GTP binding (GO:0005525), as well as pathways including Axon guidance (ko04360) and Vasopressin-regulated water reabsorption (ko04962). For Cluster 2, protein abundance declined from satiation to sleep and remained stable thereafter. The corresponding proteins were significantly enriched in ubiquitin protein ligase binding (GO:0031625) and single-stranded DNA binding (GO:0003697), along with Homologous recombination (ko03440), DNA replication (ko03030) and Mismatch repair (ko03430) pathways. In Cluster 7, protein abundance stayed steady from satiation to sleep, dropped markedly from sleep to fasting, and showed no obvious changes during the subsequent period. Related proteins were enriched in calcium ion binding (GO:0005509) and signaling receptor binding (GO:0005102), as well as Antigen processing and presentation (ko04612), lysosome (ko04142) and endocytosis (ko04144) pathways. Proteins in Cluster 9 maintained stable abundance from satiation to fasting and decreased sharply from fasting to activity. No metabolic pathways were significantly enriched for this cluster, whereas terms including GDP binding (GO:0019003) and monosaccharide binding (GO:0048029) were highly enriched.

Further comparison between protein expression trends and corresponding transcriptome data of *V. sinensis* liver revealed that proteins and genes in Cluster 7 shared similar expression patterns. Their levels remained stable from satiation to sleep, decreased markedly from sleep to fasting, and showed no obvious changes from fasting to activity. However, genes and proteins in this cluster were not enriched in identical pathways.

### 3.4. Screening Results of Rhythmic Proteins

A total of 91 rhythmic proteins were identified (Table S5), among which 34 were also DEPs. Table S5 provides detailed parameters for each rhythmic protein, including acrophase, oscillation amplitude, raw *p* values, and BH-corrected *q* values. Subcellular localization analysis showed these rhythmic proteins were mainly distributed in the nucleus, cytoplasm, membrane and mitochondrial matrix. PDZ, H15, RRM and Ig-like were the dominant conserved domains of rhythmic proteins, which differed distinctly from those of the whole proteome. This indicates that rhythmic proteins perform functions specific to circadian regulation.

### 3.5. Integrated Analysis of Transcriptome and Proteome in V. sinensis Liver

**Figure 9:**
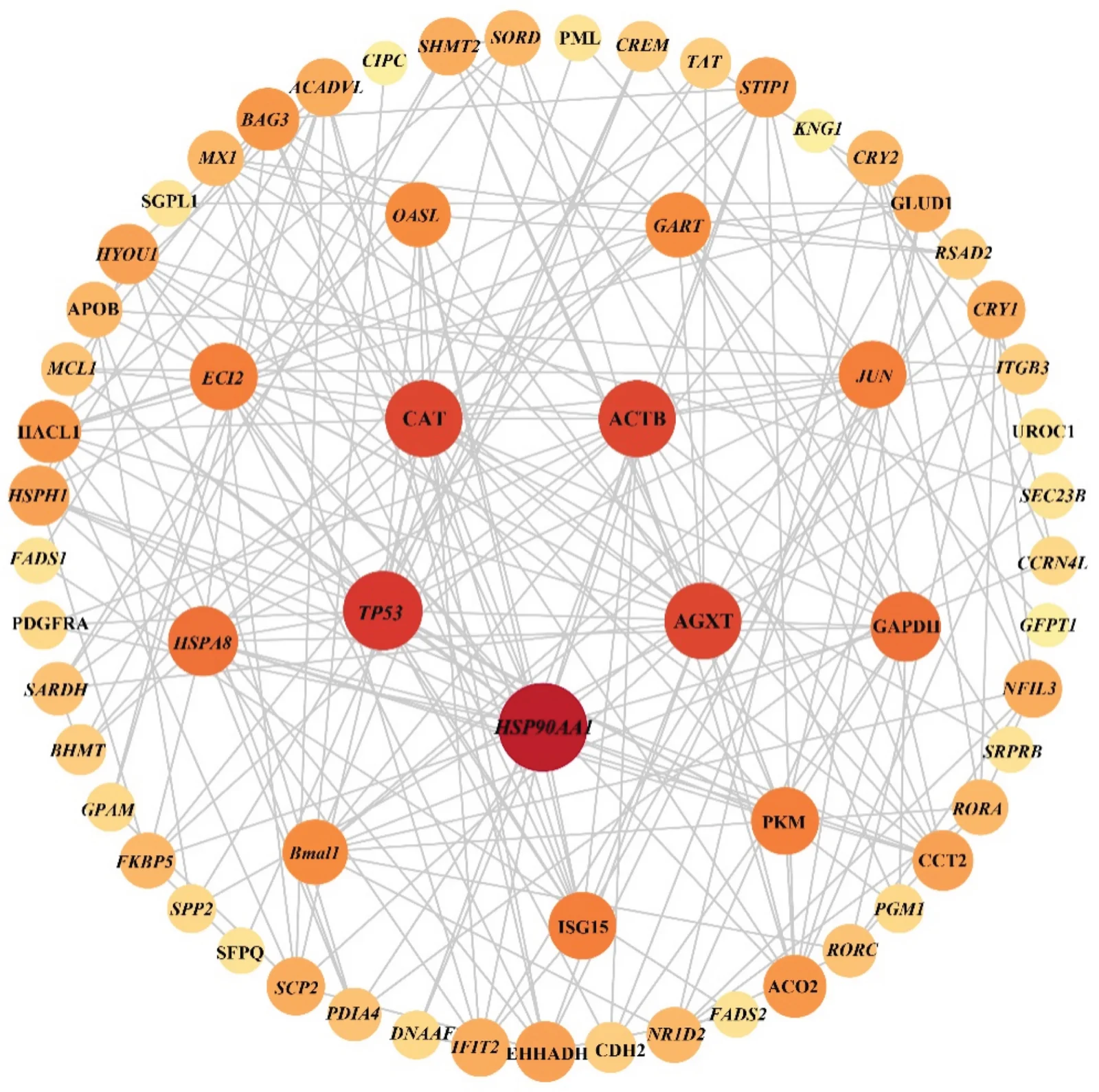
Correlation interaction network of DEGs and DEPs.

To systematically explore the correlation between gene transcription and protein translation, we compared all detected transcripts and proteins. Among the 1803 identified proteins, 667 were matched to their corresponding coding genes. Of the 91 rhythmic proteins, 34 had matched genes, and 248 out of 692 DEPs were annotated with corresponding genes. Only ten gene–protein pairs exhibited consistent rhythmic patterns, namely CYP27A1, MAOA, C9, ITGA1, FN1, HYOU1, GCSH, VTN, GST and TLN1. A correlation interaction network was constructed using DEGs and DEPs (Figure 9). Multiple key proteins closely associated with circadian rhythm regulation were screened, including SFPQ, GLUD1, HACL1, GAPDH, PKM, APOB, PML, CAT, CDH2, PDGFRA and AGXT. Meanwhile, core genes of the circadian rhythm pathway were identified, such as *CIPC*, *NFIL3*, *Bmal1*, *RORC*, *Cry1*, *Cry2*, *RORA*, *Rev-erbα* and *CCRN4L*. In this network, *HSP90AA1* showed the highest node connectivity, followed by CAT, ACTB, AGXT and *TP53.*

## 4. Discussion

Based on liver proteomic data of *V. sinensis* under four physiological states (satiation, sleep, fasting and activity), this study systematically elucidated the molecular regulatory characteristics underlying the adaptation to high energy expenditure in the liver of nocturnal bats. Analysis of subcellular localization and conserved domains revealed functional divergence between the global proteome and rhythmic proteins in bat liver, laying a molecular foundation for understanding hepatic circadian regulation. The results demonstrated that pathways related to basal metabolism and xenobiotic detoxification were persistently enriched throughout all physiological stages, while pathways including Glycolysis/gluconeogenesis and the pentose phosphate pathway were activated in a time-dependent manner. Via the regulatory mode of constitutive expression of core pathway proteins and temporal turnover of functional proteins, the liver precisely adapts to the diurnal physiological shifts between daytime rest and repair and energy-intensive nocturnal flight. Notably, a prominent decoupling between transcription and protein expression was observed. The proteome exhibited more pronounced rhythmic features than the transcriptome and metabolome, indicating that post-transcriptional regulation may play a vital role in shaping diurnal rhythms and maintaining homeostasis in bat liver. This study suggests potential diurnal adaptation strategies for metabolic and immune homeostasis in bat liver, and provides valuable data for exploring the evolutionary mechanisms of circadian adaptation in wild flying mammals.

### 4.1. Spatial Distribution and Domain Composition

Subcellular localization and conserved domain analysis were performed on the total proteins and rhythmically expressed proteins in the liver of *V. sinensis*, aiming to preliminarily elucidate the intrinsic characteristics of hepatic circadian regulation from the perspectives of spatial distribution and molecular structure. The overall identified liver proteins were mainly localized to the cytoplasm, membrane structures, nucleus and mitochondria. The predominant domains included RRM, EF-hand, Ig-like and thioredoxin, which may point to active substance metabolism, transmembrane signal transduction, calcium homeostasis and anti-oxidative capacity within bat liver. Rhythmic proteins were also predominantly distributed in the nucleus, cytoplasm, cell membrane and mitochondrial matrix, showing a distribution pattern similar to that of the total liver proteins. On the basis of these in silico predictions, we could speculate that circadian-related proteins could operate following typical spatial organizational features of hepatic cells.

In terms of conserved domain composition, obvious differences were observed between rhythmic proteins and the total hepatic proteome. The total proteome was highly enriched in domains such as EF-hand and thioredoxin, which are primarily responsible for basal homeostasis maintenance and stress response. In contrast, PDZ, H15, RRM and Ig-like were the dominant domains of rhythmic proteins. Such distinct domain preferences could suggest molecular-level functional divergence between housekeeping-associated proteins and proteins involved in circadian regulation. Most liver proteins function to sustain basal metabolism, antioxidation and routine homeostasis [6]. By virtue of their unique domain architecture, rhythm-associated proteins are preferentially involved in temporal signal transduction, chromatin modulation, RNA metabolism and rhythmic immune processes [23], enabling the liver to adapt to diurnal environmental changes and the behavioral rhythms of bats. These differences in domain composition suggest that the bat liver may harbor two sets of proteins with divergent functional properties: one set sustaining basal physiological activities and the other potentially mediating diurnal temporal regulation. Coordination between these two protein sets could hypothetically help align hepatic metabolic activities with the organismal behavioral rhythms.

### 4.2. Dynamic Enrichment and Functional Differentiation of KEGG Pathways in Bat Liver Under Distinct Physiological States

KEGG pathway enrichment analysis was conducted on DEPs in the liver of *V. sinensis* across four physiological states. The results showed that most functional pathways were not activated specifically at a single time point, but exhibited a temporal pattern of cross-enrichment across multiple stages. This indicates that to adapt to its lifestyle of diurnal roosting, intermittent feeding and high-intensity flight, the bat liver has established a molecular network featuring synergistic multiple pathways and dynamic temporal regulation. Metabolic pathways and Chemical carcinogenesis were significantly enriched throughout all four physiological stages, serving as the core backbone pathways supporting full-day physiological activities. Metabolic pathways regulate the metabolism of carbohydrates, lipids and amino acids to sustain energy supply at all physiological stages. The Chemical carcinogenesis pathway annotation primarily clusters genes encoding phase I and II xenobiotic detoxification enzymes rather than tumor-promoting factors. The continuous enrichment of the Chemical carcinogenesis pathway suggests that hepatic toxin clearance and oxidative stress neutralization may constitute constitutive rhythmic physiological processes in wild *V. sinensis*. As nocturnal insectivores with extreme flight metabolism, bats face dual chemical pressure from dietary exogenous toxins and flight-derived reactive oxygen species. The diurnal fluctuation of detoxification proteins in this pathway may enable stage-specific toxicant elimination throughout the 24 h cycle, suggesting rhythmically controlled xenobiotic detoxification as a specialized adaptive hepatic function unique to flying nocturnal mammals. This enrichment signal was validated across all physiological states and is unlikely to originate from reference annotation artifacts.

Glycolysis/gluconeogenesis, Retinol metabolism, Carbon metabolism and the pentose phosphate pathway were co-enriched during satiation, sleep and fasting, showing obvious adaptation to physiological rhythms. As core carbohydrate metabolism pathways at these stages, glycolysis and the pentose phosphate pathway share the key metabolic intermediate glucose-6-phosphate. They coordinate via substrate shunting and functional complementation to sustain daytime energy supply and antioxidant defense [24]. In the satiation state, adequate nutrients are ingested. Glycolysis is up-regulated to rapidly break down glucose for energy production, and excess carbohydrates are stored as glycogen for nutrient reserves. During sleep with no external food intake, gluconeogenesis becomes predominant. It synthesizes glucose from non-carbohydrate substrates to stabilize blood glucose and maintain basic vital activities. When energy reserves are continuously depleted during fasting, the compensatory energy supply via gluconeogenesis is further strengthened. Meanwhile, aldehyde dehydrogenases in these pathways are concurrently expressed to eliminate toxic metabolic intermediates and reduce the risk of metabolic damage [25]. The pentose phosphate pathway, activated concurrently with glycolysis, mainly generates NADPH for reductive reactions and precursors for nucleic acid synthesis [26]. On one hand, it eliminates reactive oxygen species accumulated during nocturnal flight and alleviates tissue damage caused by oxidative stress [27]. On the other hand, it provides materials for hepatocyte repair and nucleic acid metabolism, enabling metabolic partitioning between energy supply and antioxidative repair [28]. Carbon metabolism orchestrates the flux of various metabolites throughout the process and ensures the orderly linkage of carbohydrate, lipid and amino acid metabolism. Retinol metabolism coordinately modulates lipid homeostasis and inflammatory responses, further stabilizing the internal environment of the liver [29].

The biosynthesis of amino acids and drug metabolism via cytochrome P450 were persistently enriched during satiation, fasting and activity, matching the physiological demands for nutrient metabolism and exogenous defense. In the satiated state, absorbed nutrients are utilized to synthesize various amino acids for tissue growth and protein synthesis. During fasting, endogenous substances are mobilized to remodel amino acids and maintain the dynamic balance of the intracellular amino acid pool. Amino acids are catabolized to supply energy during nocturnal flight, sustaining continuous energy output under locomotion [30]. Cytochrome P450 proteins exert stage-dependent functions. They primarily degrade exogenous hazardous substances derived from food after feeding, and participate in the remodeling of endogenous lipids during fasting [31]. During high-intensity flight, these proteins neutralize oxidative metabolites and reduce the metabolic burden on the liver, enabling bats to fully adapt to complex wild habitats [32]. Tyrosine metabolism was markedly enriched during the transition from sleep and fasting to activity, indicating its involvement in the regulation of circadian phase switching. Degradation products of tyrosine such as fumarate enter the tricarboxylic acid cycle to fuel energy metabolism, which buffers energy fluctuations accompanying physiological state transitions. Additionally, this pathway potentially interacts with the regulation of redox homeostasis. Its metabolic derivatives may help mitigate elevated oxidative stress during increased activity, facilitating the smooth transition of bats from daytime rest to nocturnal flight [33].

This study also revealed that when a single pathway was enriched across multiple physiological states, its key functional proteins exhibited temporal turnover, which was most evident in the Glycolysis/gluconeogenesis pathway. Continuously enriched during satiation, sleep and fasting, this pathway maintained basic catalytic processes via the constitutive expression of proteins including ACSS1, GPI, PFKL and FBP1. Meanwhile, distinct functional enzymes showed differential expression in each stage. PDHB and FBP2 were highly expressed in the satiated state to facilitate glucose utilization and energy storage. The up-regulation of PCK2 during sleep enhanced gluconeogenesis to sustain fasting blood glucose levels [34]. In the fasting state, ADH7, ALDH3A1, ALDH2 and other proteins were predominantly expressed to support energy catabolism and eliminate toxic metabolic intermediates [35,36]. In this regulatory mode, core proteins preserve the overall framework of the pathway, while stage-specific proteins mediate functional shifts in a time-dependent manner. Rather than full activation or suppression of the entire pathway, temporal replacement of internal proteins enables adaptation to varying energy statuses. This may constitute an efficient adaptive strategy for circadian regulation in the bat liver.

Overall, the liver of *V. sinensis* forms three groups of temporally enriched pathway modules in response to diurnal physiological and behavioral changes. A flexible and stable metabolic regulatory system is established through functional coordination among pathways and stage-specific protein expression. The synergistic regulation of glycolysis and the pentose phosphate pathway is particularly critical. It balances energy supply and demand while enhancing antioxidant defense capacity, thereby revealing the molecular mechanisms underlying metabolic adaptation of nocturnal bats to their unique lifestyle.

### 4.3. Characteristics of Hepatic Circadian Regulation Mediated by Temporal Expression Patterns

Protein expression trend analysis showed that DEPs in Cluster 7 remained stable from satiation to sleep, and were significantly down-regulated during the transition from sleep to fasting. Proteins in this cluster are mainly involved in immune response, intracellular substance transport and cellular homeostasis. These findings collectively suggest rhythmic shifts in immune-related protein profiles in the liver of *V. sinensis*, with higher abundance of immune-associated proteins during rest and reduced abundance during active periods. During nocturnal flight and foraging, bats are frequently exposed to diverse environmental pathogens. Meanwhile, strenuous exercise exacerbates oxidative stress and metabolic disorders in the body [27,37]. This study found that numerous proteins involved in antigen recognition and immune activation within Cluster 7 maintained high expression levels during satiation and daytime sleep, suggesting that the bat liver may mount periodic compensatory-like immune-related molecular responses following nocturnal activity. During daytime rest, these protein profiles imply potential capacity for recognition and clearance of exogenous antigens encountered during foraging and flight, as well as immune-associated repair processes [38]. As natural hosts of various viruses, bats are reported to possess unique immune-tolerant phenotypes according to previous studies [37,39,40]. The diurnal immune-related molecular profiles observed in our dataset raise a putative hypothesis that such temporal immune patterns could potentially contribute to restraining viral replication during resting phases and avoiding over-inflammation during nocturnal activity. TAP1, a key molecule for antigen processing and transport, plays an essential role in this circadian regulation and ensures efficient and orderly antigen presentation in the liver during the daytime [41]. GO enrichment analysis revealed that the DEPs in Cluster 7 were significantly enriched in terms such as calcium ion binding and signaling receptor binding. Calcium-binding proteins including S100A8 and S100A9 mediate intracellular calcium signaling and jointly initiate immune signals as well as modulate cellular physiological states, which may contribute to shaping daytime hepatic homeostatic regulatory networks in *V. sinensis*. As a vital second messenger, calcium ions link upstream receptor signals to downstream immune responses, potentially representing one plausible mediator underlying the observed diurnal immune-related molecular rhythm in the bat liver [42].

KEGG pathway enrichment analysis indicated that proteins in Cluster 7 were significantly enriched in the lysosome, endocytosis and phagosome pathways, suggesting that hepatocytes remain highly active in damage repair and substance clearance during daytime rest. Long-term flight tends to induce protein denaturation, lipid oxidation and organelle damage. The daytime-enriched lysosomal hydrolytic machinery could facilitate the removal of damaged cellular components and metabolic wastes [27,43,44]. Whether such molecular profiles could be linked to the well-documented long-lifespan and anti-aging traits in bats awaits further experimental validation. During fasting and nocturnal activity, the overall expression of proteins related to immune defense and cellular repair in Cluster 7 decreased. This implies a transition of hepatic physiological function from daytime homeostasis maintenance to nighttime energy mobilization and metabolic energy supply, which may correspond to heightened energy-metabolic demands associated with nocturnal flight behavior. Meanwhile, the rhythmic expression of a small number of CYP-family metabolic enzymes in this cluster also assists in endogenous substance metabolism and toxin clearance in the liver during the daytime [45,46].

### 4.4. Correlation Between Transcriptome and Proteome, and Multi-Omic Characteristics of Circadian Regulation

To explore the correlation between gene transcription and protein translation in the liver of *V. sinensis*, we performed transcriptome matching analysis on all identified proteins, rhythmic proteins and DEPs. A total of 1803 liver proteins were identified, among which only 667 were matched to their corresponding coding genes. Among 91 rhythmic proteins, merely 34 had homologous gene counterparts, and only 248 out of 692 DEPs were annotated with matched genes. These results collectively reveal a low matching ratio between proteins and transcripts in bat liver. In terms of rhythmic coordination, only ten gene–protein pairs exhibited consistent diurnal rhythmic expression patterns among all matched pairs, indicating that circadian rhythms were synchronized at the transcriptional and translational levels in just a small fraction of molecules. Despite the limited number, these pairs possess important biological implications. Free from post-transcriptional noise and translational time lag, they achieve coordinated rhythmic regulation across transcription and translation throughout the diurnal cycle. We speculate that these core genes participate in nocturnal rhythm modulation, hepatic substance metabolism, oxidative stress response, cell adhesion and immune regulation. They serve as key candidate targets for further investigating diurnal metabolic reprogramming, circadian clock regulatory networks and adaptive evolutionary mechanisms in bat liver.

Meanwhile, time-series clustering results revealed that although proteins and their corresponding genes in Cluster 7 shared similar diurnal expression trends, their enriched functional pathways did not overlap. This further indicates that even with analogous temporal expression patterns, genes and proteins are involved in distinctly different physiological regulatory processes. The low matching rate, scarcity of gene–protein pairs with consistent rhythms, and divergent pathways despite similar expression trends collectively demonstrate prominent transcription–protein decoupling in the liver of *V. sinensis*.

Rhythmic analysis of all detected proteins identified 91 proteins with diurnal expression patterns, indicating periodic temporal changes in the expression of a subset of proteins in the liver of *V. sinensis*. Combined with previously reported rhythmic profiles of the transcriptome [17] and metabolome [47] in this species, we further illustrated multi-layered differences in hepatic circadian regulation at the multi-omic level. Rhythmic proteins accounted for approximately 5% of the total liver proteins, a proportion markedly higher than that of rhythmic metabolites (2%) and rhythmic transcripts (0.3%), suggesting that circadian rhythmicity was most prominent at the proteome level while extremely weak at the transcriptome level. Multi-omic studies on the liver of mammals including mice, rats and humans [12,48,49] have demonstrated that although the core circadian clock relies on transcription–translation feedback loops (TTFL) to drive rhythmic outputs, protein rhythms at the functional level are predominantly modulated by post-transcriptional regulation, translation and protein stability, rather than fluctuations in mRNA abundance alone [10].

Although the proportion of rhythmic transcripts was extremely low, the percentage of rhythmic proteins (5%) in bat liver was comparable to that reported for the liver of mice, rats and other mammals, indicating that protein-level circadian rhythms are evolutionarily conserved among mammals. Previous studies have verified that nearly half of rhythmic proteins in mouse liver do not have corresponding rhythmic mRNAs [12], and translation efficiency, protein turnover, temporal secretion and post-translational modifications are the major drivers of protein rhythmicity [50]. The pattern of weak transcriptional rhythmicity yet prominent protein rhythmicity observed in bat liver further confirms that stable protein rhythms can be established via multi-level post-transcriptional regulation even when mRNAs show no obvious oscillations, thereby orchestrating diurnal metabolism and physiological functions. The distinct differences in rhythmic molecule proportions across omics layers raise a putative hypothesis that post-transcriptional related processes could be prominent in circadian regulation in bat liver. Widespread decoupling between transcriptional and protein rhythms is common in mammalian livers, and the abundance of rhythmic proteins is not strictly dependent on periodic mRNA fluctuations [50]. This conserved pattern was also observed in bats. Such decoupling might hypothetically enable the liver to flexibly meet the demands of energy metabolism and stress defense across different sampling windows. Potential underlying mechanisms may include altered translation efficiency, protein modification, degradation, turnover and timed secretion, accompanied by limited transcriptional oscillation. The low correlation of gene–protein rhythmicity, scarce synchronized rhythmic pairs, and divergent functional pathways despite similar expression trends in Cluster 7 collectively point toward the potential importance of post-transcriptional-level processes for hepatic circadian homeostasis in bats. Our dataset lacks direct measurements of translation rates, protein turnover dynamics and post-translational modifications, and dedicated experimental assays will be essential to formally evaluate whether post-transcriptional regulation acts as the predominant regulatory layer. Likewise, further comparative multi-omics across related species will be required to assess this evolutionary scenario for adaptive tuning of diurnal regulatory strategies in this nocturnal flying mammal.

We acknowledge that the present study has several inherent limitations, and our findings require further supporting evidence from additional follow-up work for full interpretation. First, constrained by wild-animal field sampling requirements, our experimental design only includes four discrete sampling time points across the 24 h cycle. This sampling strategy enables us to characterize representative molecular signatures under natural physiological conditions, but provides relatively low temporal resolution and may overlook subtle circadian phases, expression peaks, or potential ultradian rhythmic patterns. Each physiological state was represented by three biological replicates, and natural inter-individual variation in the endogenous circadian phase among wild bats could dampen oscillation amplitudes, which may result in underestimation of the true proportion of rhythmically expressed hepatic proteins. Weak oscillatory proteins and slight expression differences may fail to reach statistical significance under this experimental design. Future studies will increase the number of biological replicates to enhance the statistical robustness of proteomic rhythmicity and differential expression analyses. Given these statistical constraints, rhythmic proteins were identified using unadjusted *p* values < 0.05. Even at this threshold, the number of rhythmic proteins remained modest. Strict multiple-testing correction would further reduce detection sensitivity under our four-point sampling scheme and risk losing genuine diurnal signals. We therefore prioritized detection sensitivity, while acknowledging that this approach bears inherent false-positive risks from multiple testing. Hence, these 91 rhythmic proteins represent suggestive candidates that require further independent experimental verification. Second, DIA proteomics possesses limited detection sensitivity for low-abundance proteins and distinct protein isoforms. This study focuses on constructing a preliminary diurnal proteomic landscape of wild bat liver under natural field conditions. In future work, we will adopt complementary enrichment strategies (such as subcellular fractionation and immune enrichment) and high-depth proteomic sequencing to capture low-abundance proteins and distinct isoforms, further refining the regulatory network of bat hepatic circadian rhythms. Third, as a non-model species, *V. sinensis* lacks a well-established reference genome. Many proteins are annotated via cross-species homology, and gene–protein pairing relied on BLAST-derived annotations against a closely related bat reference proteome. Incomplete de novo transcript assembly, tissue-specific transcript expression, and imperfect homology annotation contributed to the relatively low rate of valid gene–protein matches. The observed transcription–protein decoupling may partly originate from annotation-driven biases rather than purely biological post-transcriptional regulatory processes. Finally, this study lacked direct comparative analysis with hepatic circadian multi-omic datasets from non-flying nocturnal mammals, which would be required to rigorously separate flight-specific metabolic adaptations from general nocturnal circadian traits. Accordingly, evolutionary inferences drawn solely from our dataset should be treated with caution. Future comparative multi-omics research incorporating matched hepatic time-series profiles of non-flying nocturnal mammals will help precisely identify molecular features uniquely evolved to support bats’ sustained powered flight.

## 5. Conclusions

This study integrated DIA proteomics and transcriptomics to systematically characterize circadian regulation in the liver of *V. sinensis* across four physiological states—satiation, sleep, fasting and activity—and clarified the potential temporal metabolic and immune strategies underlying its nocturnal lifestyle and high-energy flight. The results revealed widespread decoupling between transcript and protein rhythmicity, which implies that post-transcriptional events likely participate prominently in hepatic circadian rhythms. Further targeted gene perturbation experiments are still required to formally verify whether post-transcriptional regulation acts as the core driving force behind this rhythmic uncoupling. Rhythmic fluctuations at the protein level were far more prominent than those at the transcriptional and metabolic levels, serving as the key driver of diurnal physiological transitions in the liver. Rhythmic proteins and the global proteome exhibited distinct domain compositions and functional differentiation. This enables the liver to sustain basal metabolic homeostasis while precisely responding to diurnal environmental and behavioral rhythms. Core metabolic pathways displayed temporally dynamic enrichment, governed by constitutively expressed core proteins and temporally substituted functional proteins, which ensures efficient diurnal coordination of energy supply, oxidative defense and detoxification metabolism. Temporal expression profiles suggest elevated levels of proteins associated with immune activation, antigen presentation and lysosomal damage clearance in the liver of *V. sinensis* during daytime rest. Collectively, these findings suggest potential adaptive strategies underlying hepatic rhythms in this nocturnal flying mammal at the proteomic level, and offer molecular resources for further investigations into metabolic adaptation, putative immune-tolerance traits, and longevity-associated mechanisms in bats.

## Figures and Tables

**Table 1:** The number of DEPs and the number of GO terms and KEGG pathways significantly enriched by DEPs from each comparison group.

Comparable Group	DEPs Up/Down	GO Terms (*p* < 0.05)			KEGG Pathway Up/Down (*q* < 0.05)
		Biological Process Up/Down	Molecular Function Up/Down	Cellular Component Up/Down	
Satiation vs. sleep	93/70	101/53	11/21	20/7	9/9
Sleep vs. fasting	96/76	206/65	56/30	23/2	0/18
Fasting vs. activity	187/54	74/16	13/15	17/2	27/2
Activity vs. satiation	80/322	37/166	16/40	1/16	4/12
Satiation vs. fasting	143/101	245/33	52/30	27/11	3/11
Sleep vs. activity	228/68	273/24	34/10	44/1	4/8

## Data Availability

The mass spectrometry proteomics data and processed protein abundance matrices have been deposited to the ProteomeXchange Consortium (https://proteomecentral.proteomexchange.org (accessed on 25 August 2026)) via the iProX partner repository [51,52] with the dataset identifier PXD083114.

## Supplementary Materials

The following supporting information can be downloaded at: https://www.mdpi.com/article/10.3390/biom16091289/s1, Table S1: Detailed information on DEPs identified from six pairwise comparisons. Table S2: Go terms significantly enriched by DEPs from six pairwise comparisons. Table S3: KEGG pathways significantly enriched by DEPs from six pairwise comparisons. Table S4: GO terms and KEGG pathways significantly enriched by proteins from the four significant trend clusters. Table S5: Rhythmic proteins identified by Cosinor analysis, including acrophase, amplitude, *p* values and BH-corrected *q* values.
